# Trends in educational inequalities in cause specific mortality in Norway from 1960 to 2010: a turning point for educational inequalities in cause specific mortality of Norwegian men after the millennium?

**DOI:** 10.1186/1471-2458-14-1208

**Published:** 2014-11-24

**Authors:** Bjørn Heine Strand, Ólöf Anna Steingrímsdóttir, Else-Karin Grøholt, Inger Ariansen, Sidsel Graff-Iversen, Øyvind Næss

**Affiliations:** Division of epidemiology, Norwegian Institute of Public Health, P.O. Box 4404, Nydalen, NO-0403 Oslo, Norway; Institute of Health and Society, Faculty of Medicine, University of Oslo, Oslo, Norway; Institute of Health Management and Economics, Faculty of Medicine, University of Oslo, Oslo, Norway

**Keywords:** Mortality, Education, Health inequalities, Norway, Nordic paradox

## Abstract

**Background:**

Educational inequalities in total mortality in Norway have widened during 1960–2000. We wanted to investigate if inequalities have continued to increase in the post millennium decade, and which causes of deaths were the main drivers.

**Methods:**

All deaths (total and cause specific) in the adult Norwegian population aged 45–74 years over five decades, until 2010 were included; in all 708,449 deaths and over 62 million person years. Two indices of inequalities were used to measure inequality and changes in inequalities over time, on the relative scale (Relative Index of Inequality, RII) and on the absolute scale (Slope Index of Inequality, SII).

**Results:**

Relative inequalities in total mortality increased over the five decades in both genders. Among men absolute inequalities stabilized during 2000–2010, after steady, significant increases each decade back to the 1960s, while in women, absolute inequalities continued to increase significantly during the last decade. The stabilization in absolute inequalities among men in the last decade was mostly due to a fall in inequalities in cardiovascular disease (CVD) mortality and lung cancer and respiratory disease mortality. Still, in this last decade, the absolute inequalities in cause-specific mortality among men were mostly due to cardiovascular diseases (CVD) (34% of total mortality inequality), lung cancer and respiratory diseases (21%). Among women the absolute inequalities in mortality were mostly due to lung cancer and chronic lower respiratory tract diseases (30%) and CVD (27%).

**Conclusions:**

In men, absolute inequalities in mortality have stopped increasing, seemingly due to reduction in inequalities in CVD mortality. Absolute inequality in mortality continues to widen among women, mostly due to death from lung cancer and chronic lung disease. Relative educational inequalities in mortality are still on the rise for Norwegian men and women.

## Background

Why socioeconomic health inequalities persist and even widen in the modern welfare states of Western Europe has been denoted a paradox [1]. Even in the Nordic countries, where the welfare state regime is generous with a focus on equality, there have been widening inequalities in the last decades, both on an absolute and a relative scale [2–4]. The Nordic countries differ from other welfare state regimes [5], and stand out from the other Western European countries with lower level of income inequality, lower prevalence of poverty, generous social security benefits and a health care system mostly publicly funded [6].

Previously, we reported widening educational inequalities (both relative and absolute) in mortality among middle aged Norwegians in the period 1960–2000 [2]. Cardiovascular mortality was the main driver behind the inequalities the whole period. The increase in inequality during the period was driven by cardiovascular mortality in men, and by deaths due to chronic lower respiratory tract diseases and lung cancer in women. We have also reported a flattening of educational inequalities in life expectancy in men after year 2000, while inequalities continue to increase in women [7]. Using national cause specific mortality data, we build on our previous work to examine if the educational inequalities in mortality in Norway have continued to increase after the millennium, and which causes are important regarding the educational inequalities.

## Methods

Data was compiled by Statistics Norway and based on individual records from the Norwegian Cause of Death Registry and the National Education Data Base, and linked using the personal identification number unique to every Norwegian resident. Number of person years and numbers of deaths were summed up for each year 1961 to 2010 for those aged 45 to 74 years that individual year. Five decades were constructed by adding up person years and deaths between January 1^st^ and December 31^st^ in the periods 1961–1969, 1970–1979, 1980–1989, 1990–1999, and 2000–2009. The study included 708,449 deaths and 62.3 million person years (Table 1). Data was stratified by gender, five year age groups and in the three educational groups basic, secondary and tertiary. Basic education comprised those with nine years of schooling (seven years in the 1960s), the secondary group comprised those with primary and secondary education (10–12 years), and the tertiary group comprised those with post-secondary education (13+ years; college and university). Data on education was almost complete with only 0.9–1.3% missing all decades. The education stratification for all our analyses, and all decades, is based on Statistics Norway’s revision of 2006, when the definitions were changed to be more in line with international criteria [8]. The new criteria are stricter regarding reaching secondary education, and with the new classification the basic education group increased from 19.0% to 32.8% in 2005, while the secondary education group was reduced from 56.7% to 42.4% [8]. Tertiary education had only a minor change from 24.3% to 24.8%. In this report we have re-analyzed the data from all periods according to the new criteria. Overall, the tertiary educated group increased steadily in size from 6% in the 1960s to 25% in the 2000s in men and from 3% to 22% in women (Table 1).

**Table 1:** **Background table**

	1960s	1970s	1980s	1990s	2000s
Educational level	**Number of deaths**				
**Men**					
Basic	57558	68257	60498	43298	27688
Secondary	19725	31488	37090	35028	30839
Tertiary	3489	5532	6723	8161	9013
Total	80772	105277	104311	86487	67540
**Women**					
Basic	43539	45396	38394	30054	20526
Secondary	8105	13906	15885	17212	17450
Tertiary	1419	1888	2326	3204	4758
Total	53063	61190	56605	50470	42734
	**Number of person years (%)**				
**Men**					
Basic	3318547 (66)	3367182 (56)	2726452 (47)	2145114 (34)	1836114 (25)
Secondary	1437370 (29)	2136814 (36)	2381412 (41)	2907905 (46)	3584177 (50)
Tertiary	282055 (6)	494505 (8)	699045 (12)	1203933 (19)	1813014 (25)
Total	5037972 (100)	5998501 (100)	5806909 (100)	6256952 (100)	7233305 (100)
**Women**					
Basic	4081861 (75)	4214395 (65)	3544338 (57)	2766130 (42)	2232559 (31)
Secondary	1149819 (21)	1944846 (30)	2257147 (36)	2867124 (44)	3420863 (47)
Tertiary	186186 (3)	314531 (5)	464903 (7)	898462 (14)	1639013 (22)
Total	5417866 (100)	6473772 (100)	6266388 (100)	6531716 (100)	7292435 (100)
	**Mortality rate (per 100 000 py), age adjusted**				
**Men**					
Basic	1618.1	1742.2	1713.1	1557.4	1302.3
Secondary	1506.9	1512.6	1423.1	1170.3	883.1
Tertiary	1320.2	1224.1	1041.5	803.9	574.3
Total	1569.7	1617.7	1513.3	1232.5	912.1
**Women**					
Basic	938.1	871.4	785.7	779.9	746.4
Secondary	767.5	689.2	630.3	569.5	495.9
Tertiary	738.5	587.1	529.8	434.2	360.7
Total	895.0	802.9	710.7	640.0	542.2

Number of deaths, person years and mortality rate by educational level, period and gender for age 45–74 years.

Causes of death were categorized according to the International Classification of Diseases using the 8th and ninth revision until 1996 and the 10th revision thereafter. Deaths were grouped in seven groups (ICD-10 codes): cancer of lung (C32-C34), other cancer (C00-C32, C35-C97), cardiovascular diseases (I00-I99), suicide (X60-X84), external causes (excluding suicide) (V01-Y89), chronic lower respiratory tract diseases (J40-J47), and other causes. Bridging between versions of ICD-8 to ICD-10 was done using Eurostat’s European Short List of Causes of Death [9].

### Statistical methods

Age adjusted mortality rates per 100,000 person years were calculated using the direct method and European standard population weights (ISD1976) [10]. Two regression based indices of inequality were used to take into account the change in educational distribution over the five decades [11]. When studying mortality over time, and in the situation with falling mortality rates across all educational strata, absolute inequalities may be stable over time, while relative inequalities are increasing. Because of this we included both an absolute and a relative measure of inequality, which is recommended when studying health inequalities [12]. The Slope Index of Inequality (SII) is an epidemiological measure of absolute inequality in health applicable to ordinal socioeconomic variables [12], and the corresponding measure on the relative scale is The Relative Index of Inequality (RII). When we refer to results regarding absolute mortality inequalities in the paper it is the SII we refer to, and regarding relative mortality inequalities we refer to the RII. These indices take into account the distribution of educational groups and are useful when comparing social inequalities over time or between countries. These indices can be interpreted as the increased risk of dying related to be on a lower level in the educational hierarchy. To calculate these indices the educational groups were ordered from lowest to highest level of education and each group was assigned a so-called Ridit-score (which is the percentage of the population with higher socioeconomic position, here education) [12]. The Poisson regression model was used to estimate the two inequality indices with 95% confidence intervals, regressing the age adjusted number of deaths (age adjusted rate multiplied with the person years) on the Ridit-score (continuous) and decade (categorical variable) and their interaction terms, specifically for each gender:

ln(age adjusted number of deaths)=ln(personyears)+a+b*ridit

+c*decade1970+d*decade1980+e*decade1990+f*decade2000

+g*ridit_decade1970+h*ridit_decade1980+i*ridit_decade1990+j*ridit_decade2000 +error,

where ln(person years) is the natural logarithm for person years, a is the intercept, b is the coefficient for the Ridit-score, c-f are the coefficients for the decades and g-j are the coefficients for the interaction terms Ridit-score by decade.

The SII (for the 1960s) was calculated post-regression (using the nlcom-command in Stata) as a nonlinear combination of the estimated coefficients as: SII_1960 = 100,000*exp([a + b]/a). For other decades, for example 1970, SII was estimated as: SII_1970 = 100,000*exp([a + b + c + g]/[a + c]). A similar approach was used to estimate SII for the other decades using the appropriate decade-variables and the interaction term Ridit-score by decade. To test for differences in SII between decades, combinations of the above estimates were used, for example the difference between 1970 and 1960: SII_1970 - SII_1960. By including the interaction term Ridit-score by decade it was possible to test for difference in SII between decades. For a test of the trend over the whole period a similar model as specified above was used, with decade as a continuous variable labelled 0, 1, 2, 3, 4, where 0 represented 1960s, 1 represented 1970s, and so on. A similar approach as described above was used to estimate the RII, and the estimation was based on the exact same model as used for the SII. The post-regression-calculation is a bit simpler for the RII than the SII because the RII is just the exponentiated Ridit-score-coefficient. Thus, the RII for the 1960s is RII_1960 = exp([b]), and for the subsequent periods the relevant interaction terms are added. Stata version 12 was used for all analyses.

The study has been performed with the approval of The Regional Committee for Medical and Health Research Ethics in Norway, and is in compliance with the Helsinki Declaration.

## Results

In men, absolute educational inequalities (SII) in total mortality flattened out after year 2000 – in fact there was a 2% non-significant (p = 0.56) drop between the periods 1990–99 and 2000–09 (calculated as: (SII_2000 – SII_1990)/SII_2000 = (1035–1050)/1050 (Table 2). This happened after steadily, significantly increasing absolute inequalities all the way back to the 1960s. In women there was a drop in absolute inequalities from the 1970s to 1980s, but thereafter a steady increase, also after year 2000 (p-value < 0.01). Relative inequalities (RII) increased sharply in men and women in all decades (p < 0.001), except from 1970s to 1980s in women where inequalities were stable (Table 2).

**Table 2:** **Absolute and relative educational inequalities in mortality in Norway over five decades, by gender**

	Men		Women	
Decade	Absolute inequality* (95% CI)	Absolute change in inequality* from previous period	Absolute inequality* (95% CI)	Absolute change in inequality* from previous period
1960s	313 (265, 361)		376 (336, 416)	
1970s	626 (583, 670)	313 (p < 0.01)	427 (395, 458)	51 (p = 0.05)
1980s	838 (796, 879)	211 (p < 0.01)	364 (336, 391)	−63 (p < 0.01)
1990s	1050 (1013, 1087)	212 (p < 0.01)	495 (470, 519)	130 (p < 0.01)
2000s	1035 (1005, 1066)	−14 (p = 0.561)	551 (529, 572)	56 (p < 0.01)
*P-value for linear trend*	*0.018*		*0.025*	
	**Men**		**Women**	
**Decade**	Relative inequality** (95% CI)	Change in relative inequality from previous period***	Relative inequality** (95% CI)	Change in relative inequality from previous period***
1960s	1.21 (1.18, 1.26)		1.52 (1.46, 1.59)	
1970s	1.46 (1.42, 1.50)	0.18 (p < 0.01)	1.69 (1.63, 1.76)	0.11 (p < 0.01)
1980s	1.71 (1.67, 1.76)	0.16 (p < 0.01)	1.67 (1.61, 1.73)	−0.01 (p = 0.59)
1990s	2.24 (2.18, 2.30)	0.27 (p < 0.01)	2.14 (2.07, 2.22)	0.25 (p < 0.01)
2000s	2.95 (2.86, 3.03)	0.27 (p < 0.01)	2.72 (2.62, 2.82)	0.24 (p < 0.01)
*P-value for linear trend*	*<0.001*		*0.013*	

*Slope Index of Inequality, SII. The number can be interpreted as the difference in absolute risk (unit is deaths per 100,000 person years) of dying related to be on a lower level in the educational hierarchy compared to be at the top.

**Relative Index if Inequality, RII. The number can be interpreted as the mortality incidence rate ratio for those in the lower level in the educational hierarchy relative to those at the top.

***Change in ln(RII). Since RII is estimated on a log-scale, we investigate change using ln(RII) as this converts it to linear scale.

**Figure 1:**
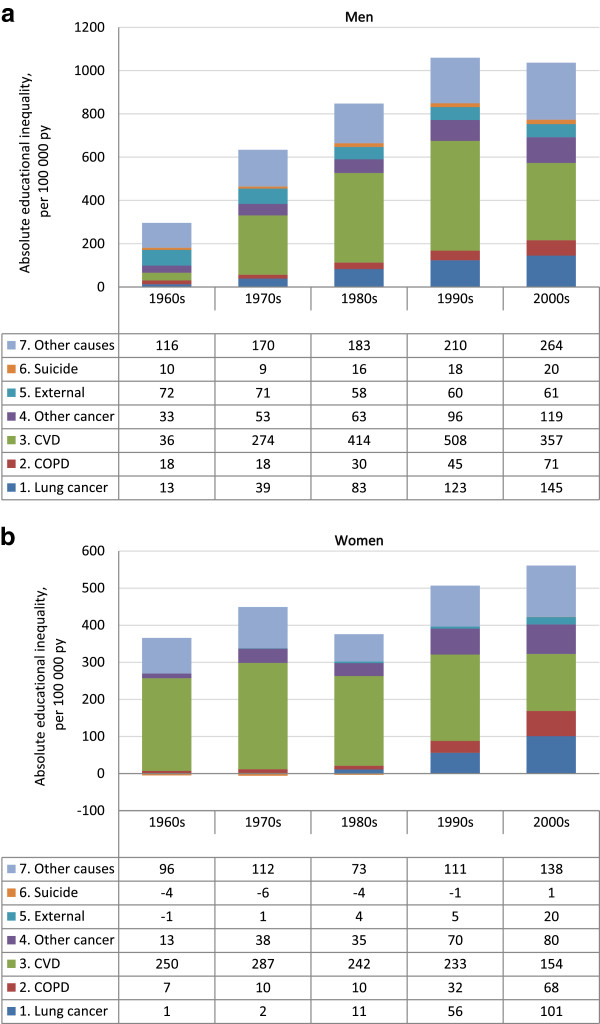
**Absolute inequality (SII) in total and cause specific mortality over five decades (1961–2009), age adjusted. a**. Men. Absolute educational inequality* in cause specific mortality for Norwegians aged 45–74 years over five decades (1961–2009), age-adjusted. The total height of the bars represent absolute inequality in all-cause mortality (per 100,000 person years). **b**. Women. Absolute educational inequality* in cause specific mortality for Norwegians aged 45–74 years over five decades (1961–2009), age-adjusted. The total height of the bars represent absolute inequality in all-cause mortality (per 100,000 person years).

Another noteworthy change in the post millennium decade from previous decades was the large drop in absolute inequality in CVD mortality among men; the SII for CVD dropped from 508 to 357 deaths per 100,000 (test of change in absolute inequalities: p < 0.001) (Figure 1a). Since the 1960s CVD has been the largest driver of the absolute educational inequalities in total mortality in both men and women. Overall, there was a drop in CVD mortality for all educational groups the last decades, in both men and women, and in the last decade this drop was greatest in the lowest educational group (Table 3). The impact of educational inequalities in CVD mortality to the total absolute mortality gap by education was reduced from 48% to 34% in men, and from 46% to 27% in women (Figures 1a and b). In men, death inequalities for all other causes increased in the same period (Figure 1a). In women there was a steady decrease in absolute CVD inequalities since the 1970s, and this decrease continued after millennium (test of change in absolute inequalities between 1990–99 and 2000–09: p < 0.001) (Figure 1b).

**Table 3:** **Cause specific mortality rates (in 7 cause specific groups) by gender, educational level and period for the ages 45–74 years**

	1960s	1970s	1980s	1990s	2000s
Educational level					
1. Lung cancer mortality rate (per 100 000 py), age adjusted					
**Men**					
Basic	57.1	88.3	121.8	140.7	144.7
Secondary	54.1	76.0	95.0	97.4	91.4
Tertiary	40.1	52.1	55.1	53.7	45.1
Total	50.4	72.1	90.6	97.2	93.8
**Women**					
Basic	9.2	15.3	30.1	61.8	93.8
Secondary	7.9	14.9	26.0	38.3	49.6
Tertiary	14.1	12.5	20.4	24.3	26.1
Total	10.4	14.2	25.5	41.5	56.5
2. COPD mortality rate (per 100 000 py), age adjusted					
**Men**					
Basic	21.3	30.5	35.4	45.0	58.5
Secondary	14.9	25.1	25.9	30.9	31.3
Tertiary	9.1	13.9	11.5	13.2	11.7
Total	15.1	23.2	24.3	29.7	33.9
**Women**					
Basic	6.5	8.8	12.8	28.2	50.4
Secondary	3.9	5.0	8.5	15.1	22.1
Tertiary	3.1	3.0	6.0	7.5	7.3
Total	4.5	5.6	9.1	16.9	26.6
3. CVD mortality rate (per 100 000 py), age adjusted					
**Men**					
Basic	804.4	886.6	843.7	667.2	404.4
Secondary	809.0	794.2	707.9	483.7	259.7
Tertiary	708.7	635.6	500.0	305.2	156.3
Total	774.0	772.1	683.8	485.4	273.5
**Women**					
Basic	423.4	377.5	304.3	244.2	157.9
Secondary	313.2	258.3	201.9	146.9	90.7
Tertiary	296.0	203.1	148.9	89.2	52.9
Total	344.2	279.6	218.4	160.1	100.5
4. Other cancer mortality rate (per 100 000 py), age adjusted					
**Men**					
Basic	296.1	318.8	321.4	321.4	289.2
Secondary	283.7	299.9	303.2	290.4	253.3
Tertiary	265.9	271.0	262.9	246.7	201.7
Total	281.9	296.6	295.8	286.2	248.0
**Women**					
Basic	272.5	282.6	274.8	273.9	248.3
Secondary	268.0	268.8	260.4	248.1	213.9
Tertiary	258.4	243.1	246.6	218.7	190.5
Total	266.3	264.9	260.6	246.9	217.6
5. External causes mortality rate (per 100 000 py), age adjusted					
**Men**					
Basic	79.1	85.1	77.3	67.9	65.1
Secondary	51.4	56.8	55.0	45.2	40.5
Tertiary	36.6	41.7	37.2	26.6	23.2
Total	55.7	61.2	56.5	46.6	43.0
**Women**					
Basic	19.4	20.8	20.5	17.7	25.5
Secondary	19.4	20.8	18.8	15.9	14.7
Tertiary	22.9	19.0	17.6	13.6	12.4
Total	20.6	20.2	19.0	15.7	17.5
6. Suicide mortality rate (per 100 000 py), age adjusted					
**Men**					
Basic	21.8	26.8	36.2	31.3	28.5
Secondary	16.7	22.1	27.8	23.3	18.6
Tertiary	18.6	22.6	27.4	19.3	14.4
Total	19.0	23.8	30.5	24.6	20.5
**Women**					
Basic	5.8	8.0	12.5	10.2	9.5
Secondary	7.2	10.3	13.6	10.4	8.6
Tertiary	10.3	14.2	16.7	11.3	9.1
Total	7.7	10.8	14.3	10.6	9.1
7. Other causes mortality rate (per 100 000 py), age adjusted					
**Men**					
Basic	302.5	304.4	273.9	273.0	293.0
Secondary	254.1	237.1	203.7	190.3	173.8
Tertiary	225.2	186.3	143.1	128.1	111.6
Total	260.6	242.6	206.9	197.1	192.8
**Women**					
Basic	172.9	157.8	128.7	140.2	154.1
Secondary	132.2	110.7	99.6	91.8	91.1
Tertiary	115.4	90.8	72.9	66.1	59.1
Total	140.2	119.9	100.4	99.4	101.4

Furthermore an important finding is the substantial widening in absolute inequalities in lung cancer and chronic respiratory disease in the post millennium decade among women (test of change in absolute inequalities between 1990–99 and 2000–09: p < 0.001, Figure 1b). In women, this widening gap for these causes is a continuation of a significantly widening trend since the 1980s (test of change in absolute inequalities: p < 0.001 between each decade 1980s to 2000s).

In women, educational inequalities in deaths due to lung cancer and chronic respiratory diseases combined constituted 30% of the total absolute mortality inequality in the post millennium decade, and were thereby more important than CVD (27%) regarding the total mortality gap by education (Figure 1b). In comparison, in all previous decades inequalities in deaths from CVD constituted most of the total mortality gap in women (69% in the 1960s, 65% in the 1970s and 1980s, 46% in the 1990s, Figure 1b).

Also in men, absolute inequalities in lung cancer and chronic respiratory diseases increased in the last decade 2000–2009, and this increase was significant each decade since the 1970s (lung cancer and chronic respiratory diseases combined constituted 10% of absolute inequalities in total mortality in the 1960s, 9% in the 1970s, 14% in the 1980s and 16% in the 1990s, Figure 1a). In men, these causes combined constituted 21% of the total absolute mortality inequality during 2000–2009, but CVD was still more important (34%), as it was in the previous three decades (43% in the 1970s, 49% in the 1980s and 48% in the 1990s) (Figure 1a).

Absolute educational inequalities in external causes and suicide were stable over time in men, and constituted in combination 8% of the total absolute male mortality inequality during 2000–2009. In women the corresponding figure was 4%. Absolute educational inequalities in other cancers and other causes increased after millennium and constituted 37% and 39% of the total educational inequalities in mortality in men and women respectively.

## Discussion

For the first time in five decades absolute mortality inequalities have stopped to increase among men, even with a tendency toward a drop the last decade. This levelling out in inequalities among men is mainly due to a large and significant drop in CVD mortality inequalities. Due to falling mortality rates across all educational strata, relative educational inequalities in mortality are still on the rise for Norwegian men and women, as is the case in North, West and East of Europe [13]. For women this increase also holds for absolute inequalities, in line with our previous reports [2, 7]. The impact of educational inequalities in CVD on the total mortality gap was substantially reduced in both men and women the last decade. In women this decrease in inequality started already in the 1990s, but for men, the fall in CVD mortality inequalities was first seen in the last decade.

The overall rates of CVD mortality have decreased markedly from 1970s, and particularly marked from the middle of 1980s for ischemic heart disease, especially in men [14]. In both men and women, this fall in CVD mortality was more pronounced in the higher educational groups until year 2000, while from 2000 the fall was greatest in the lowest educational group, leading to flattening educational inequalities in the last decade. For women this CVD-driven drop of inequality was not out-weighted by the large increase in inequalities in deaths from lung cancer and chronic respiratory diseases, resulting in a continued increase of the gap in total mortality between those at the top and bottom in the educational hierarchy. Thus, even if CVD mortality inequalities continued to narrow in women the last decade, inequalities in total mortality increased. A population-based regional Norwegian survey found that the educational trend in serum cholesterol levels weakened significantly between 1994 and 2008 [15]. If these results are nationally representative, this trend predicts cessation of the increase in the educational gap in CVD mortality, consistent with our findings in men.

As the decrease in coronary heart disease mortality in the Western countries during the last decades of the last century is related to better treatment [16], more coronary heart disease patients are living with rather than dying from coronary heart disease and thus are at risk of dying of other risk factor-related diseases, especially long term smoking-related diseases such as chronic obstructive pulmonary disease and cancer. Also, smoking cessation affects cardiovascular health more immediately than cancers [17].

Norway has the position as the country in Europe with the largest educational inequalities in smoking [18], and in Norway educational inequalities in smoking have been on the rise since the 1970s [19], with a possible fall after 2009 [20]. It seems that smoking matters more in Norway regarding educational inequalities in total mortality compared with other European countries [21–23]. In Finland there are large educational inequalities in female smoking, and smoking was the main driver behind the increased inequalities in female mortality during 1971–2010, while among men smoking was not as important [24]. Similar findings have been reported in New-Zealand [25]. In South-European countries there are smaller educational inequalities in smoking, which is also reflected in smaller differences in smoking-related deaths [21]. Also in US there seems to be other causes than the smoking, which are driving the increased educational inequalities in mortality [26].

Lung cancer mortality started declining in adult men during the mid 1990s, after steady increase since the 1950s, while in women lung cancer mortality incidence started to increase later, around 1970, and did not decline until 2010 [14, 27]. In line with the world-wide tobacco epidemic described by Lopez et al. in 1994 [28], higher educated people are being affected mostly in the epidemic’s early stages [27, 29]. Those in the higher educational groups were the first to pick up smoking and also the first to abstain or quit [27], especially among women [30]. In men, the prevalence of daily smoking started decreasing around 1970, from 52% in 1973 to 19% in 2010, while in women the corresponding figures were 32% and 19% [31]. Prevalence has continued to decrease to 15% in men and 14% in women in 2013.

## Conclusions

In men absolute educational inequalities in total mortality stopped increasing in the post millennium decade seemingly due to a reduction in inequalities in CVD mortality. In women, inequalities (both on the absolute and relative scale) in mortality have continued to widen in Norway after millennium, mostly due to causes of death such as lung cancer and COPD. Educational inequalities in total mortality seem to be moving from CVD mortality being the single most important driver to a more heterogeneous pattern where deaths from several non-communicable diseases contribute. Inequalities in mortality should be monitored closely in the coming years.
